# Fuzzy-Based Sensor Fusion for Cognitive Load Assessment in Inclusive Manufacturing Strategies

**DOI:** 10.3390/s25113356

**Published:** 2025-05-27

**Authors:** Agnese Testa, Alessandro Simeone, Massimiliano Zecca, Andrea Paoli, Luca Settineri

**Affiliations:** 1 Department of Management and Production Engineering, Politecnico di Torino, Corso Duca degli Abruzzi 24, 10129 Torino, Italy; 2 School of Mechanical, Electrical and Manufacturing Engineering, Loughborough University, Ashby Rd, Loughborough LE11 3TU, UK

**Keywords:** sensor fusion, cognitive load, inclusive manufacturing, neurodiversity, assembly

## Abstract

In recent years, the need to design inclusive workplaces has grown, particularly in manufacturing contexts where high cognitive demands may disadvantage neurodiverse individuals. In manufacturing environments, neurodiverse workers often experience difficulties processing standard instructions, increasing cognitive load and errors and reducing overall performance. This study proposes a methodology to assess cognitive load during assembly tasks to support workers with dyslexia. A multi-layer fuzzy logic framework was developed, integrating physiological, environmental, and task-related data. Physiological signals, including heart rate, heart rate variability, electrodermal activity, and eye-tracking data, were collected using wearable sensors. Ambient conditions were also measured. The model emphasizes the Reading dimension of cognitive load, critical for dyslexic individuals challenged by text-based instructions. A controlled laboratory study with 18 neurotypical participants simulated dyslexia scenarios with and without support, compared to a control condition. Results indicated that a lack of support increased cognitive load and reduced performance in complex tasks. In simpler tasks, control participants showed higher cognitive effort, possibly employing overcompensation strategies by exerting additional cognitive resources to maintain performance. Support mechanisms, such as audio prompts, effectively reduced cognitive load, highlighting the framework’s potential for fostering inclusive practices in industrial environments.

## 1. Introduction

Over the years, discussions about workplace inclusivity have evolved from raising awareness and improving physical accessibility to creating environments that also support mental wellbeing and accommodate neurodiverse needs [1]. Inclusive employers now strive to foster spaces that are not only physically accessible but also promote mental health and value neurological differences [2]. Recognising neurodiversity as a spectrum is crucial; individuals possess a range of characteristics that vary widely and evolve over time. For employers and designers, this means prioritising workplaces that address physical, cognitive, and emotional needs [3]. Involving neurodiverse individuals in design and evaluation can transform workplaces from merely accessible spaces into supportive environments for a wide range of mental and neurological conditions. By integrating diverse perspectives, organisations can create settings where employees thrive and unlock the full potential of the workforce [4].

Dyslexia, a learning disability that affects the development of literacy skills such as reading, writing, and spelling, presents unique challenges. Despite similar educational opportunities, individuals with dyslexia often struggle significantly more than their peers [5]. Estimating its prevalence is challenging due to overlaps with other conditions such as alexia or educational disadvantages; however, it is generally estimated that dyslexia affects around 10% of the population, with figures varying based on diagnostic criteria, awareness levels, and assessment methods [5]. Adults with dyslexia often encounter difficulties in written tasks, including handwriting, organisation of written work, multitasking, note-taking, and following sequential instructions. Negative past experiences further discourage many from disclosing their dyslexia [6], yet these individuals often demonstrate strengths in creativity, social skills, resourcefulness, and entrepreneurship [5].

In modern manufacturing, continuous improvement, innovation, and the effective utilisation of diverse talents are essential. Integrating neurodiverse talent has emerged as a key strategy to enhance creativity, productivity, and quality in industries facing 21st-century challenges [7]. Neurodiverse employees contribute unique cognitive strengths, offering fresh insights and creative solutions to complex problems. Their exceptional attention to detail and sustained concentration are particularly valuable in manufacturing, where precision and accuracy are critical for quality control and minimising errors [8].

However, despite these strengths, neurodiverse individuals often face significant challenges that can impact their full participation in the workforce, such as difficulties in emotional regulation, reduced environmental mastery, low coping skills, and obstacles in self-determination. These challenges can manifest in the manufacturing context through difficulties in adapting to fast-changing environments, handling complex social interactions within teams, and managing work-related stress or sensory overload commonly found in industrial settings. Therefore, fostering a truly inclusive workplace means not only recognising the strengths of neurodiverse individuals but also implementing supportive strategies that accommodate their specific needs [9].

The inclusion of neurodiverse individuals expands the pool of skills available, enabling better task assignments, improved team dynamics, increased productivity, enhanced employee engagement, and a more attractive workplace culture that reduces turnover and attracts top talent. Embracing neurodiversity also promotes continuous learning and adaptability, which are vital qualities in rapidly changing manufacturing environments. The benefits extend beyond equity and diversity, positioning organisations to drive innovation, improve operational efficiencies, and enhance product quality for a more inclusive and successful future [10].

Recent years have witnessed a substantial increase in research contributions containing the term “neurodiversity”. While research output remained relatively low for much of the previous decade, a notable rise began around 2020, followed by a sharp acceleration in subsequent years. This trend has continued to intensify, with the number of publications steadily increasing in recent years. This growth tends to indicate an increased awareness of neurodiversity in both the literature and industrial practices [11].

The term neurodiversity was coined in the 1990s, but it has taken almost twenty years for the word to become so widely used [12]. At least part of this recent upsurge can be explained by a concomitant increase in emphasis on identifying the strengths of neurodiverse people, their inclusion in the workforce and their quality of life. This emerging academic interest underlines the relevance of the topic and ensures its importance in current research and debates related to inclusion and diversity issues in different fields [13].

In such context, this research aims to support the inclusion of neurodiverse individuals within the manufacturing sector by addressing barriers associated with cognitive workload and stress. Industrial environments are typically fast paced and cognitively demanding, which may present particular challenges for neurodiverse employees as human–robot interaction becomes increasingly prevalent [14]. To promote equitable working conditions, it is essential to gain a clear understanding of how cognitive load develops and to identify periods characterised by elevated stress or mental fatigue [15].

This study investigates the assessment of cognitive load during assembly tasks by examining physiological, environmental, and task-related variables. The objective is to dynamically estimate cognitive load during task execution in an industrial environment, with the aim of identifying reliable indicators that can support the development of inclusive strategies and reduce stress in cognitively demanding contexts. In particular, this research explores how a multimodal sensor fusion approach can be employed for real-time cognitive load estimation, whether reading-related cognitive effort varies across different support conditions in dyslexia-simulated tasks, and to what extent robotic assistance may offer advantages over human support in mitigating cognitive demands.

## 2. Literature Review

This section reviews the literature on cognitive load assessment, with approaches ranging from individual sensor-based methods to sensor fusion, human–robot collaboration, and machine learning integration. Such approaches are examined to overcome the limitations of single-modality techniques and demonstrate how multifaceted strategies contribute to a more sophisticated cognitive load modelling.

### 2.1. Single Technologies

Cognitive workload assessment has received growing research attention, particularly through the use of physiological signals to better understand mental effort and emotional distress [16]. Among the most widely studied methods are heart rate variability (HRV), electrodermal activity (EDA), pupillometry, and electrocardiography (ECG).

HRV is a key indicator of autonomic nervous system activity and has been used extensively to monitor changes in cognitive workload. It is commonly analysed across three frequency bands: very low frequency (VLF, 0–0.04 Hz), low frequency (LF, 0.04–0.15 Hz), and high frequency (HF, 0.15–0.4 Hz). Increased cognitive demand has been shown to reduce HF power and elevate LF power, suggesting a shift towards sympathetic dominance and reduced parasympathetic control [17]. Overall, HRV decreases in response to heightened mental effort, likely due to increased sympathetic activation or parasympathetic withdrawal [18,19].

Additionally, EDA and, in particular, the galvanic skin response (GSR), measures fluctuations in skin conductance associated with sweat gland activity and is considered a robust marker of sympathetic arousal. It comprises both tonic (slow-varying) and phasic (rapid) components [20]. The literature shows how EDA is widely used to track cognitive load, attention, and emotional processing, and it remains a preferred physiological measure for assessing stress responses [21,22]. Baseline condition selection is critical for accurate EDA analysis. Krebl et al. [23] demonstrated that passive visual tasks such as watching calming videos or observing fixation points yield more stable baselines than minimally demanding games.

Pupillometry, via eye-tracking technologies, has emerged as a sensitive tool for monitoring cognitive workload. Metrics such as fixations, saccades, and pupil dilation offer real-time insights into visual attention and mental effort. Skaramagkas et al. [24] highlight the application of machine learning techniques to eye-tracking data, enhancing the detection of both cognitive load and emotional arousal.

Finally, ECG provides direct measures of cardiac activity and serves as an accessible tool for assessing mental workload, particularly in dynamic environments. It supports the derivation of HRV metrics and complements other physiological indicators, enabling a more comprehensive assessment of cognitive states [25].

### 2.2. Sensor Fusion

Recent advances in physiological monitoring have demonstrated that integrating multiple biosignals can improve the accuracy and robustness of cognitive workload assessment. Sensor fusion approaches, which combine data from distinct modalities, address limitations inherent in single-sensor systems and enhance resilience in dynamic, real-world environments [26].

Affanni [27] contributed to this field by developing a wearable, wireless sensor system capable of monitoring ECG and EDA during active tasks. Its capacity to reduce motion artefacts while transmitting real-time data makes it particularly suitable for physically dynamic collaborative scenarios. Comparable systems have been validated in industrial settings, where wearable biosensors provide robust physiological inputs to detect operator strain [28].

Ayres et al. [29] examined whether physiological measures can be used to differentiate intrinsic cognitive load, finding that HRV and pupillometry serve as reliable reflections of cognitive load with discriminant validity that allows differentiation between task difficulty levels. Their research suggests that HRV and pupil size can be effectively employed to review the mental loads assigned to complex learning and performance tasks.

Vanneste et al. [30] investigated a multimodal framework combining EDA, electroencephalography (EEG), and electrooculography (EOG). Their analysis revealed that physiological markers such as the skin conductance response rate, alpha power, and blink rate correlated significantly with self-reported workload, explaining nearly 23% of the variance. Similarly, Ma et al. [31] employed eye-tracking and ECG sensors to assess HRV and pupillometric indices across four levels of task complexity. Their results confirmed that experts displayed consistently lower cognitive load across conditions, demonstrating the utility of combining cardiac and ocular markers.

More recently, Liu et al. [32] proposed a multiview sensor fusion framework incorporating EEG, ECG, EDA, EOG, and eye movement data to predict cognitive load during multitask scenarios. Their model achieved over 80% classification accuracy and highlighted the complementary value of different signal types.

Additionally, Kuttala et al. [33] demonstrated that fusing hierarchical features from ECG and EDA using convolutional neural networks improved stress classification across multiple datasets.

### 2.3. Human–Robot Collaboration and Cognitive Workload Assessment

The integration of collaborative robots (cobots) into industrial environments presents both challenges and opportunities for cognitive workload assessment. As human operators increasingly share responsibilities with robotic systems, continuous monitoring of cognitive state becomes essential for maintaining safety, efficiency, and user wellbeing [34].

Rajavenkatanarayanan et al. [35] introduced RoboAssist, a real-time framework combining ECG and EDA data to classify cognitive load during collaborative assembly. Their system demonstrated reliable detection of high-load states in a study involving 25 participants, providing a foundation for cognitively adaptive robotic systems. Similar approaches have shown that combining physiological signals through sensor fusion can significantly improve recognition accuracy in real-world conditions [36].

Cognitive load also shapes interpersonal factors such as trust in automation. Research has shown that varying cognitive demands influence how humans evaluate and rely on robotic partners during shared tasks. A high workload can increase perceived dependency, while a low workload may heighten sensitivity to errors, highlighting the importance of balancing system support with operator autonomy [37]. These dynamics are especially relevant in hybrid collaboration contexts, where trust and cognitive burden interact closely [38].

### 2.4. Integration with Machine Learning for Enhanced Assessment

The integration of machine learning (ML) techniques into physiological data analysis marks a substantial step forward in cognitive workload assessment. By uncovering complex, non-linear relationships across multimodal biosignals, ML enables a more precise and scalable evaluation of mental effort. Initial evidence supports the discriminative capacity of physiological signals. Recent studies have shown that ML classifiers, particularly support vector machines and deep neural networks, can achieve high accuracy in cognitive state recognition. For example, using the STEW dataset, Safari et al. [39] achieved 89.53% classification accuracy by combining EEG-based brain connectivity features with feature selection strategies. Similarly, Wang et al. [40] applied frequency-domain features and ML classifiers to distinguish cognitive load across simulated driving conditions. Afzal et al. [41] further extended this line of work by introducing a deep gated neural network (DGNN) as the core of a cognitive workload monitoring system capable of capturing dynamic mental state fluctuations.

### 2.5. Implications for Neurodiverse Populations in Industrial Settings

Understanding how to assess cognitive load in neurodiverse populations has become increasingly important as human–robot interactions grow more prevalent in industrial contexts. The structured and predictable behaviour of cobots, which operate according to predefined routines, may align particularly well with the preferences of individuals with certain neurodevelopmental conditions. This alignment holds potential for designing more inclusive work environments that accommodate diverse cognitive profiles [42].

In smart factory settings, physiological signals including EEG and functional near-infrared spectroscopy (fNIRS) have been used to predict worker stress under varying task complexities, suggesting their applicability in neurodiverse user groups as well [14]. These techniques provide objective, real-time insights that may outperform traditional subjective assessments, particularly when verbal reporting is limited.

Meta-analytic findings also support the use of EEG and GSR as sensitive markers of cognitive workload in collaborative assembly contexts, although technical constraints remain a consideration [43].

Cognitive load refers to the amount of mental effort spent to process information and perform tasks. In complex working conditions, such as manufacturing, understanding and managing cognitive load would be a step towards improvement in productivity and efficiency and general wellbeing [31]. On the contrary, an increased cognitive load will generate lower performance, higher error rates, and enhanced levels of stress, especially across diverse cognitive profiles [44]. For an effective estimation of cognitive load, not only should the aspect of physiological conditions be considered but also those related to external environmental surroundings [44].

While there is a growing body of literature contributions on neurodiversity and inclusion in a variety of fields, there are currently very few targeted studies that address the difficulties faced by neurodiverse people in manufacturing, particularly when it comes to human–robot collaboration. Such a gap is represented by the lack of frameworks to promote inclusion in terms of how stress and cognitive load affect neurodiverse workers during manufacturing tasks [42].

To address this gap, this research proposes a framework to estimate the cognitive load in real-time by fusing sensor data from physiological variables, the working environment, and the manufacturing process.

## 3. Research Framework and Methodology

**Figure 1:**
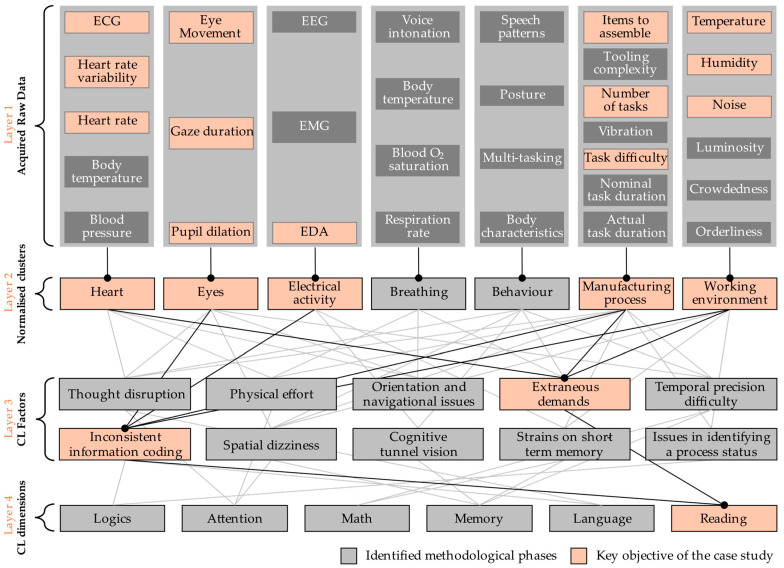
Cognitive load modelling adapted from [45].

The research framework illustrated in Figure 1 consists of four distinct layers, each designed to analyse and quantify cognitive load contributions using different inputs.

### 3.1. Layer-Wise Structure

#### 3.1.1. Layer 1: Acquired Raw Data

In this phase, various physiological, environmental, and process-related variables are collected using diverse sensing units. Physiological data include heart rate (HR) [29], HRV [19,26], representing changes in the intervals between successive heartbeats, and ECG, which records the electrical activity of the heart over time [19,26]. Other physiological variables include eye movements, fixation duration, and pupil dilation, all tracked using an eye tracker [46]. Pupil dilation serves as an indicator of noradrenergic activity and cognitive arousal. In addition, EDA is measured to assess skin conductance, an important physiological marker of stress and cognitive load. The GSR, which represents the phasic component of EDA, detects rapid changes in skin conductance associated with fluctuations in cognitive load [47].

Environmental variables, including ambient temperature, humidity, and noise levels, are recorded [48]. Additionally, task-related parameters such as the number of tasks performed, task difficulty [49], and the number of items to be assembled are monitored. The number of items to be assembled is a normalised parameter that increases with the number of components required to complete a product.

Ultimately, the output of this layer yields a comprehensive collection of raw sensory data that will be further processed to analyse the physiological, environmental, and process-related variables.

#### 3.1.2. Layer 2: Normalised Variable Clusters

The variables acquired in the previous layer are first normalised against baseline measurements recorded under resting conditions [50]. Heart rate data normalisation accounts for both tachycardia and bradycardia by dividing each sample by the mean heart rate, with values significantly deviating from ones indicating higher cognitive load [51]. Eye and electrical activity data are normalised similarly, with higher values indicating increased cognitive load [52]. Post-normalisation, values are scaled to ranges of 0–2 for heart rate, ECG, and HRV and 1–2 for eye and electrical activity data. Environmental parameters (temperature, humidity, and noise) are also normalised: humidity is scaled to 0–1, and noise is categorised as low (0), medium (0.5), or high (1). Manufacturing process variables (number of items, operations, and task difficulty) are normalised similarly, with difficulty mapped to 0, 0.5, or 1 [53].

The normalised data are categorised into distinct clusters based on their characteristics and impact on cognitive load [50]: the Heart Cluster (HR, HRV, and ECG) monitors autonomic nervous system responses [54]; the Eye Cluster (eye movements, fixation duration, and pupil dilation) reflects attention and cognitive processing [55]; the Electrical Activity Cluster consists of EDA, measuring sympathetic nervous system responses to cognitive and emotional stress [56]. The Manufacturing Process Cluster captures aspects of task complexity, quantified through the number of tasks, task difficulty, and the number of items to be assembled [57]. Lastly, the Working Environment Cluster includes environmental factors such as temperature, humidity, and noise levels, which influence both worker wellbeing and task performance [58].

Layer 2 organises Layer 1 data into coherent clusters using fuzzy set theory. Subsequently, each connection in the flowchart diagram (Figure 1) represents a fuzzy inference mechanism, progressively integrating simpler variables into more complex ones [45]. This hierarchical approach effectively supports adaptive risk assessment in human–cyber–physical systems associated with Operator 5.0, referring to human operators enhanced by smart, digital technologies enabling improved collaboration with automation systems [59].

#### 3.1.3. Layer 3: Cognitive Load Factors

This layer identifies the cognitive load factors that are directly influenced by the normalised data. Layer 3 acts as a bridge, interpreting how the collected variables translate into real-world cognitive challenges faced by workers.

The cognitive load factors include [45,60]:

1. *Thought disruption*: Refers to interruptions that break the continuity of mental processing, leading to fragmented cognitive flow and reduced task performance;
2. *Physical effort*: Captures the mental burden associated with physically demanding tasks, such as manual operations or sustained postural exertion;
3. *Orientation and navigation problems*: Encompass the cognitive load required to interpret spatial layouts or navigate through physical or procedural environments;
4. *Extraneous demands*: Mental effort imposed by inefficient tasks or system design, such as unclear instructions, redundant steps, or poorly organized interfaces. These demands consume attention and processing resources without contributing to task goals, often hindering performance and increasing the likelihood of error;
5. *Temporal precision difficulty*: Refers to increased cognitive effort when tasks demand strict timing coordination or synchronization with external events;
6. *Inconsistent information coding*: Reflects the mental effort required to reconcile or interpret data presented in varying formats or terminologies;
7. *Spatial dizziness*: Involves disorientation due to complex or rapidly changing spatial environments, affecting situational awareness;
8. *Cognitive tunnel vision*: A narrowing of attention where the individual fixates on certain elements of a task while neglecting others, often induced by high workload or stress;
9. *Strains on short-term memory*: Reflects overload in working memory capacity due to complex tasks or environmental distractions, impairing temporary information storage and manipulation;
10. *Issues in identifying process status*: Represents challenges in monitoring and understanding the current state of a process, often due to poor system feedback or unclear indicators.

#### 3.1.4. Layer 4: Cognitive Load Dimensions

In layer 4, the cognitive load factors identified in the previous layer are mapped onto six cognitive load dimensions relevant to manufacturing operators:

11. *Logic*: The ability to engage in logical reasoning and process information for decision making;
12. *Attention*: The ability to maintain focus and awareness in the face of distractions or task demands;
13. *Mathematics*: Challenges associated with performing calculations or engaging in tasks that require numerical skills;
14. *Memory*: The recall and application of information in the short term;
15. *Language*: Understanding and using language to carry out tasks;
16. *Reading*: The ability to read and interpret written material, distinguishing relevant information from irrelevant information.

Although the present study focuses on the Reading dimension, the structure of the model allows for future implementation and evaluation of the remaining dimensions, which may be relevant in other neurodiversity-related contexts.

### 3.2. Data Processing

Physiological signals, including cardiac activity and electrodermal responses, undergo case-specific preprocessing using filtering techniques to remove noise and artefacts. Similarly, eye-tracking measures, such as pupil diameter and gaze patterns, are processed to ensure signal integrity. Environmental and task-related variables are also considered.

To enable comparison with baseline values, each sample obtained during the experimental tests is normalised by dividing it by the mean of the corresponding baseline signal. This normalisation provides a relative measure of deviation from the baseline. The resulting signals are then used as inputs to a fuzzy logic system, which models cognitive load using a multi-layer inference framework.

### 3.3. Fuzzy Logic Modelling

The fuzzy modelling approach in this study is designed to interpret cognitive load by integrating multiple physiological, environmental, and process-related inputs through a multi-layer inference system [61].

Each input variable is characterised by a set of membership functions, derived from distributions and thresholds derived from the relevant literature [62], which classify its influence on cognitive load into distinct fuzzy levels. During the fuzzification process, crisp input values are converted into fuzzy sets. A fuzzy inference system, also informed by the existing literature [63], models the interactions among inputs to estimate cognitive load, producing a fuzzy output. The defuzzified output ranges from 0 to 1, as determined by the design of the fuzzy inference system. In this framework, 0 corresponds to low cognitive load, 0.5 to medium load, and 1 to high load, according to the structure of the rules and membership functions. Such a progressive approach allows for the model to aggregate layer-wise outputs to the final output representing the various dimensions of cognitive load.

The categorization of cognitive load into three levels (low, medium, and high) was selected as a trade-off between computational complexity and practical implementability in real-time applications. This classification aligns with existing fuzzy-based cognitive load models that adopt a similar three-level granularity to ensure interpretability and computational efficiency [61,63]. The design of the membership functions was informed by physiological thresholds and value distributions reported in the literature, particularly those applied to HR, HRV, ECG, EDA, and eye-tracking metrics [62,64].

While the overall fuzzy output range was normalised to [0–1] and structured into three categories (low, medium, and high) for interpretability, the membership functions were not uniform across the system. Each subsystem was designed with specific input variables and associated membership functions tailored to the nature and dynamic range of the corresponding physiological or behavioural signals. The parameters and number of MFs varied depending on the complexity and sensitivity of the input and were defined based on both empirical distributions from the dataset and the relevant literature [61,63].

This study investigates the reading-related dimension of cognitive load, modelled through a multi-layer fuzzy logic framework. Raw input variables are first normalised and clustered in the second layer and then combined in the third layer into higher-order factors, including *Extraneous Demands* (reflecting environmental, manufacturing, and physiological elements) and *Inconsistent Information Coding* (capturing visual and physiological discrepancies). These factors inform the fourth-layer construct of *Reading*, which forms the core focus of this research. Reading is a critical factor in assessing cognitive load among individuals with dyslexia, a neurodevelopmental condition marked by difficulties in reading and language processing. For this population, text-based tasks such as interpreting assembly instructions, forms, or labels impose elevated cognitive demands [64]. This study addresses the challenges faced by dyslexic workers during such tasks and highlights the risk of cognitive overload when information is not presented in a way that supports their visual processing needs [65].

## 4. Case Study

The proposed framework has been implemented and validated in a laboratory-scale case study. Duplo blocks were used to simulate an assembly task analogous to a small-scale manufacturing environment, chosen for their simplicity and safety while still allowing effective assessment of cognitive load [66].

**Figure 2:**
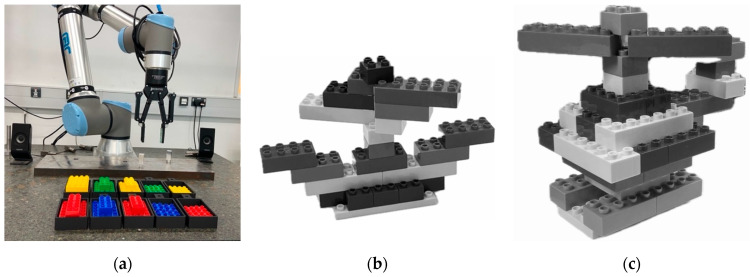
Experimental setup: (**a**) construction blocks and collaborative robot; (**b**) Construction 1 with Duplo blocks for Test 1; (**c**) Construction 2 with Duplo blocks for Test 2.

The experimental setup involved participants performing two consecutive assembly tests with Duplo blocks. In Test 1, participants completed 12 tasks by following on-screen instructions to build Construction 1 (Figure 2b). In Test 2, they carried out 11 tasks to build Construction 2 (Figure 2c), again guided by instructions displayed on the screen. At the beginning of each test, participants were shown the grayscale images for 3 s to give a general impression of the target construction while minimising the amount of information provided.

Two experimental conditions were defined: Human Assistance and Robot Assistance. In the Human Assistance condition, a person provided Duplo blocks to the participant upon request (Figure 2a) (prompted by on-screen instructions), whereas, in the Robot Assistance condition, a collaborative robot supplied the requested blocks [67]. Participants selected the pieces via a touchscreen interface, and the robot subsequently delivered them.

### 4.1. Participants

A total of 18 participants, all under 30 years of age, were recruited through an open call at Loughborough University. Only individuals without any diagnosed neurodiversity-related conditions were included. Demographic data such as gender or educational background were not collected, in accordance with restrictions set by the approved ethical protocol. However, the sample can be considered relatively homogeneous, as all participants were university students, within a narrow age range, and without diagnosed neurocognitive conditions. Ethical approval was secured from Loughborough University, and participants provided full informed consent before taking part. The recruitment of neurotypical participants was adopted due to ethical and institutional constraints that currently limit the direct involvement of neurodiverse individuals in experimental protocols. This approach, though limited in ecological validity, is consistent with established methods in preliminary cognitive load research [45,68]. Each participant completed two tasks under the experimental conditions reported in Table 1. The sequence of tests was randomised to ensure that all combinations of conditions were covered in different orders across participants to counterbalance potential order effects, thereby reducing biases in the analysis. An initial baseline phase lasting 3 min was conducted before each task, during which participants remained seated and attempted to minimise movement.

### 4.2. Data Acquisition

To continuously and non-invasively assess physiological and behavioural indicators of cognitive demand in the workplace, a range of wearable sensing devices were employed. These tools captured moment-to-moment biosignals aligned with cognitive responses under task conditions.

Heart rate data were obtained via the Polar OH1 optical sensor, worn on the left upper arm. It recorded data at 1 Hz and transmitted signals via Bluetooth to the Polar Flow app for real-time monitoring. In parallel, the EcgMove 4 device provided high-resolution (1024 Hz) single-channel ECG recordings via disposable electrodes placed on the chest, also enabling the calculation of heart rate variability metrics such as RMSSD. Electrodermal activity was measured with the EdaMove 4, which sampled skin conductance at 32 Hz using exosomatic Ag/AgCl electrodes worn on the non-dominant hand. Eye-tracking data were captured using the Tobii Pro Glasses 3, a binocular system operating at 100 Hz, equipped with a Full HD scene camera (106° field of view), infrared illuminators, and a gyroscope. Outputs included gaze coordinates, pupil diameter, eye movement types, and timestamps.

Participants completed two construction-based tests using Duplo blocks of varying sizes and colours, stored in custom 3D-printed boxes arranged in front of a robotic system. Before each test, participants viewed a black-and-white illustration of the target structure. Test 1 consisted of 12 tasks involving timed actions and graphical interpretation, while Test 2 included 11 sequential tasks under similar conditions. Each task consisted of a single instruction displayed on the screen, guiding the participant through the corresponding assembly step. Although participants followed step-by-step instructions, they were allowed to revisit previous steps if needed. Each test was limited to 15 min and ended automatically either upon task completion or when the time expired. The difficulty of each task is presented in Table 2 for Test 1 and in Table 3 for Test 2, along with the number of items to be assembled, defined as the number of Duplo blocks used for that specific task, and the number of operations required to complete it.

**Figure 3:**
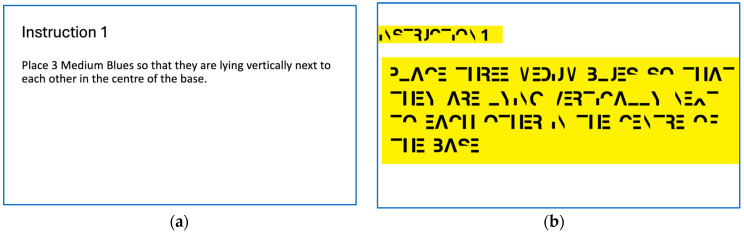
Example of instructions: (**a**) Control scenario; (**b**) Simulated dyslexia scenario.

Three experimental scenarios were designed to assess participants’ responses under varying cognitive demands. In the Control scenario (Figure 3a), instructions were presented in a standard font, establishing a baseline without added reading difficulty. In the Dyslexia with support scenario, instructions were presented in a dyslexia-simulating font [69] illustrated in Figure 3b, and participants could request pre-recorded audio prompts. This setup simulated the type of support often provided to individuals with dyslexia [68]. In the Dyslexia without support scenario, the same font was used, but no audio prompts were available, thereby increasing the reading load.

Prior to each session, participants were equipped with four sensors: a Polar OH1 heart rate monitor on the left arm, an EcgMove 4 sensor attached to the chest with disposable electrodes, an EdaMove 4 sensor on the non-dominant hand, and Tobii Pro Glasses 3 for eye tracking. Once all devices were fitted and recording began, participants completed a 3-min baseline period in a resting state to stabilise physiological measurements. This was followed by instructions and Test 1, after which participants took a 3-min break before continuing to Test 2.

Environmental data (temperature, humidity, and noise) and the start/end times of each test were also recorded and synchronised with all the other sensor signals for subsequent analyses. In addition, scene videos recorded by the eye tracker were manually reviewed to note precise moments when participants advanced from one instruction to the next.

### 4.3. Cognitive Load Assessment

HR, ECG, and HRV data were collected during baseline, Test 1, and Test 2 conditions. HR and HRV signals were upsampled to match the ECG sampling rate (1024 Hz), ensuring temporal alignment. Data from Tests 1 and 2 were normalised using baseline means, constraining values within a range of 0–2 for consistent comparisons. ECG signals were filtered with a 50 Hz notch filter, followed by a second-order low-pass Butterworth filter at 110 Hz and an additional low-pass filter at 0.05 Hz to remove residual noise. Concerning the fuzzy process settings, the heart rate and ECG utilised five Gaussian membership functions, while HRV employed a Pi-shaped function (low) and two Gaussian functions (medium, high). Inputs were mapped onto three output levels, i.e., low, medium, and high, representing the cognitive demand.

Pupil diameter (left and right) and gaze data (eye movement type and gaze duration) were recorded at 100 Hz, eliminating the need for resampling. Data from Test 1 and Test 2 were normalised against baseline means, scaling values between 1 (baseline) and 2 (high cognitive load), and assigning numerical values to fixation (2) and saccade (1) events. Cognitive load was assessed using a fuzzy logic model with Gaussian membership functions. Finally, a low-pass filter (0.05 Hz) was applied to the resulting signals (right eye and left eye) to minimise high-frequency noise.

Three vectors (baseline, Test 1, and Test 2) were collected for EDA and filtered using a second-order Butterworth low-pass filter at 1 Hz to reduce high-frequency noise. Following the same procedure as for pupil diameter, test samples were normalised by dividing by the baseline mean and scaled between 1 (baseline) and 2 (high cognitive load). A further low-pass filter at 0.05 Hz was applied to retain relevant low-frequency variations. The filtered signals were then input into the fuzzy logic system for cognitive load assessment.

Environmental parameters (temperature, humidity, and noise) were recorded for each experimental session. Humidity was normalised by dividing by 100, placing values on a 0–1 scale, while noise levels (“low”, “medium”, and “high”) were coded numerically as 0, 0.5, and 1, respectively. These data were assessed via fuzzy logic: temperature was represented using five Gaussian membership functions centred at 15 °C, 19 °C, 22 °C, 25 °C, and 28 °C; humidity similarly used five functions centred at 0.1, 0.3, 0.5, 0.7, and 0.9; noise levels were directly mapped to their numerical categories.

The values for the number of items and operations were normalised by dividing each by the maximum observed value per test, and task difficulty was numerically mapped to 0 (low), 0.5 (medium), or 1 (high). These normalised data served as inputs to a fuzzy inference system, which assigned a cognitive load level to each task.

In the third layer, the Extraneous Demands integrate three inputs: Heart, Manufacturing Process, and Working Environment. The latter two were resampled to match the 1024 Hz sampling rate of the Heart signal, and all inputs were normalized to a 0–1 range. In this layer, three Gaussian membership functions were used, centred at 0.1 (low), 0.5 (medium), and 0.9 (high).

The other third-layer factor is Inconsistent Information Coding, which integrates Eye, Electrical Activity, Manufacturing Process, and Working Environment signals. Eye data (originally 100 Hz) were downsampled to 32 Hz to match the Electrical Activity frequency, ensuring minimal loss of critical information due to the low-frequency content of the data. The Manufacturing Process and Working Environment signals were similarly adapted. All inputs were normalised within a 0–1 interval and processed through three Gaussian membership functions centred at 0.1 (low), 0.5 (medium), and 0.9 (high), capturing the progressive increase in cognitive load.

The fourth and final layer, Reading, integrates the outputs from Extraneous Demands and Inconsistent Information Coding. To ensure temporal alignment, the Inconsistent Information Coding data were resampled to match the Extraneous Demands frequency. Once aligned, these inputs were processed through Gaussian membership functions centred at 0.1 (low), 0.5 (medium), and 0.9 (high) cognitive loads.

## 5. Results and Discussion

**Figure 4:**
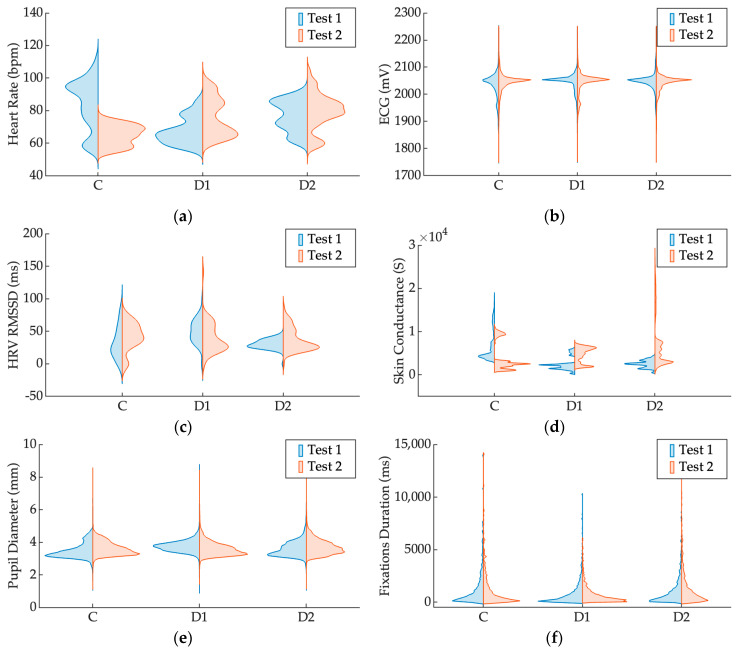
Violin plots representing the distribution of raw physiological and eye-tracking data across the three experimental conditions: Control (C), Dyslexia with support (D1), and Dyslexia without support (D2). Each plot compares values collected during the two reading tasks: Test 1 (blue) and Test 2 (orange). The figure includes the following: (**a**) heart rate; (**b**) ECG amplitude; (**c**) heart rate variability (HRV); (**d**) electrodermal activity (EDA); (**e**) pupil diameter; and (**f**) fixation duration.

Concerning the physiological variables, the violin plots illustrated in Figure 4 show distinct physiological responses across the three scenarios, i.e., Control, Dyslexia with support, and Dyslexia without support for Test 1 and Test 2, respectively.

The heart rate data (Figure 4a) show clear distinctions among groups, particularly between the Control and Dyslexia scenarios. The Control group consistently shows narrower distributions with lower median values, indicating more stable physiological arousal. In contrast, both Dyslexia groups exhibit broader distributions, suggesting greater variability in heart rate responses. A slight increase in heart rate from Test 1 to Test 2 is evident across all scenarios, particularly pronounced in the Dyslexia without support scenario.

For ECG measurements, shown in Figure 4b, while median values remain similar across scenarios, the Dyslexia with support group displays a more pronounced variability compared to Control and Dyslexia without support, particularly in Test 2. The Control scenario remains the most consistent across both tests.

In the analysis of HRV (RMSSD) shown in Figure 4c, the Dyslexia without support group distinctly differs from the other scenarios, showing a noticeably broader distribution in Test 2, indicative of higher physiological variability or stress response. The Control group displays the narrowest distribution across both tests, suggesting greater physiological stability.

EDA data (Figure 4d) further highlight differences, with Dyslexia with support exhibiting consistently elevated skin conductance levels across both tests, compared to the Control and Dyslexia without support groups. Notably, variability increases markedly during Test 2, suggesting a possible increased cognitive or emotional load in the second session.

Analysis of pupil diameter distributions in Figure 4e reveals clear, consistent distinctions among the three experimental scenarios. The Dyslexia without support group exhibits an increased pupil diameter, reflected by higher median values and greater variability compared to the Control and Dyslexia with support groups. This pattern is consistent across both Test 1 and Test 2. Conversely, the Dyslexia with support and Control groups show similar and consistently lower pupil diameters. Differences between Test 1 and Test 2 across scenarios are minimal, highlighting the stability of pupil dilation responses upon task repetition.

Finally, the distributions for gaze event durations shown in Figure 4f demonstrate highly consistent patterns across the three experimental scenarios (Control, Dyslexia with support, and Dyslexia without support) as well as between Test 1 and Test 2. Median fixation durations and variability remain comparable in all scenarios, indicating stable visual processing characteristics regardless of the experimental scenario.

These results are presented for descriptive purposes only. No inferential statistical analyses were performed due to the limited sample size and the exploratory nature of the study. Therefore, differences observed across conditions should be interpreted as preliminary trends rather than statistically validated effects.

### 5.1. Cognitive Load Results

**Figure 5:**
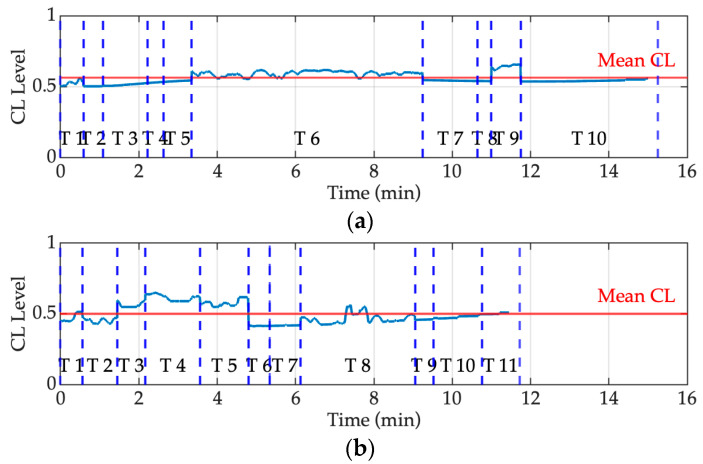
Cognitive load levels for the Reading output for Participant 1 in (**a**) the Dyslexia with support scenario and (**b**) the Control scenario.

In Figure 5, cognitive load is segmented by tasks (T) to track cognitive variations over time and tasks. Blue vertical dashed lines indicate the start of each task (T1 to T11 for Test 1 and T1 to T12 for Test 2), while the red line represents the average cognitive load across the entire session. The output is scaled in the [0–1] range, with values closer to 1 indicating higher cognitive effort.

A comparison between the Dyslexia with support and Control scenarios reveals significant differences in cognitive effort across tasks. Specifically, in the Dyslexia with support scenario (Figure 5a), the cognitive load remains relatively stable throughout most tasks, with a modest rise around Task T6. However, towards the later tasks (T9), there is a slight rise in cognitive load, indicating a potential accumulation of effort despite the support.

In contrast, in the Control scenario (Figure 5b), cognitive load is more variable across tasks but remains lower overall. Small peaks are observed, particularly between Tasks T3 and T5 and around T8, possibly due to task content or attention shifts.

Mean cognitive load is higher in the Dyslexia scenario (approximately 0.57) compared to the Control scenario (approximately 0.50), indicating that reading with dyslexia requires greater sustained effort, even when support is available.

The output of the reading dimension of CL from all participants was collected and analysed by categorising the data into the three test scenarios. Specifically, the output of the cognitive load signal of the Reading fuzzy output was segmented into the 12 tasks for Test 1 and the 11 tasks for Test 2. Each output was segmented into distinct tasks to extract the corresponding portion of the cognitive load for each task in each participant.

**Figure 6:**
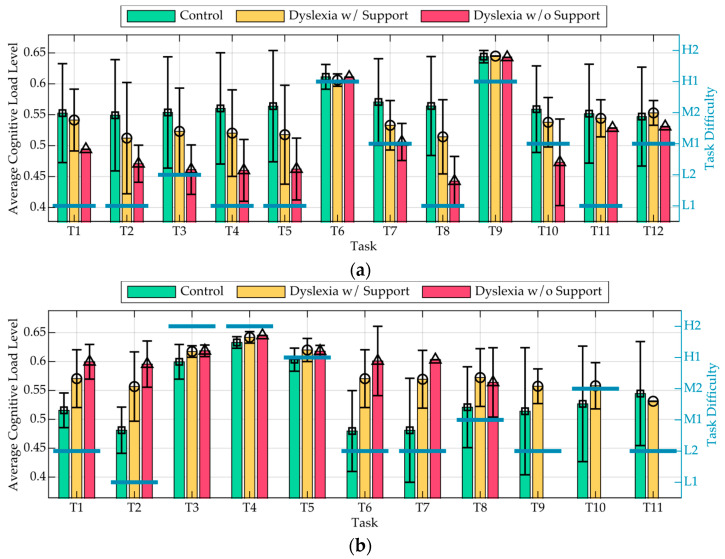
Average cognitive load levels per task and test scenario (Control, Dyslexia with support, and Dyslexia without support) for the Reading output in (**a**) Test 1 and (**b**) Test 2. Vertical black error bars indicate the standard deviation. Task difficulty levels are shown as horizontal blue lines, labelled from L1 (Low 1) to H2 (High 2).

Figure 6 illustrates the variation in cognitive load across tasks and test scenarios. Specifically, Figure 6a presents group-level means and standard deviations for each task in Test 1, corresponding to the Control group, and the Dyslexia with and without support conditions. Similarly, Figure 6b reports results for Test 2. Vertical black error bars represent standard deviations, indicating intra-group variability. Task difficulty was categorised into five levels, Low 1 (L1), Low 2 (L2), Medium 1 (M1), Medium 2 (M2), and High 1–2 (H1, H2), and is displayed using horizontal blue bars annotated with the corresponding labels.

From the charts, it can be observed how cognitive load follows a similar trend across scenarios, with fluctuations corresponding to varying task difficulty. However, notable differences emerge in the magnitude of these fluctuations.

The cognitive load graphs reveal that, in Test 1 (Figure 6a), the Control scenario exhibits a higher cognitive load than the Dyslexia without support scenario, which is counterintuitive, as individuals with dyslexia typically face greater reading difficulties. A possible explanation may be related to the relative ease of Test 1: if the test was not particularly challenging, individuals with simulated dyslexia might have adopted compensatory strategies that reduced their cognitive load, while the Control participants, being more proficient readers, may have exerted additional effort to maintain high accuracy, increasing their cognitive demand. This interpretation is supported by the standard deviation bars, which show greater variability in the Control group compared to the Dyslexia conditions. This suggests that cognitive effort in the Control scenario was less consistent across participants, possibly reflecting individual differences in reading strategy or task engagement [70].

In Test 2 (Figure 6b), the Dyslexia without support scenario consistently exhibits the highest cognitive load, particularly in certain tasks where it diverges more sharply from the other scenarios. This suggests that reading without assistance imposes greater cognitive effort, leading to increased strain. The Dyslexia with support scenario remains closer to the Control group, with reduced variability in cognitive load, indicating that assistance helps stabilise effort and reduce peaks of strain. This is further reflected in the standard deviation values, which remain lower in the Dyslexia conditions. Task 9 stands out as the most cognitively demanding across all scenarios, as shown by sharp peaks. This suggests that the nature of this specific task is inherently more challenging, regardless of the presence of dyslexia or external support. Conversely, towards the final tasks, cognitive load tends to decrease, potentially indicating adaptation to the task structure or a reduction in cognitive effort required. The Dyslexia without support group exhibited a notable drop in cognitive load between Tasks 7 and 8, despite the increase in task difficulty. This counterintuitive trend suggests the presence of a give-up phenomenon [71], where participants, recognising the imminent time limit, disengaged from the task rather than continuing to exert cognitive effort.

**Figure 7:**
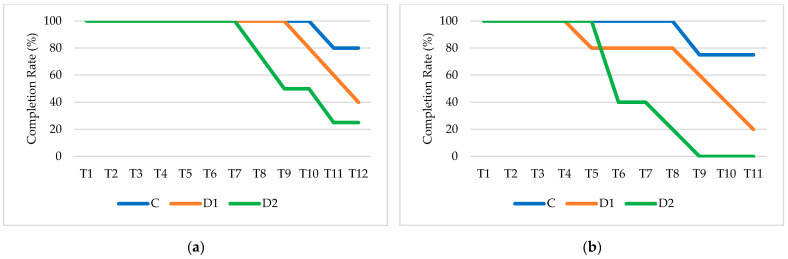
Participant completion rate through the experiment across the test scenarios (Control (C), Dyslexia with support (D1), Dyslexia without support (D2)) for the Reading output in (**a**) Test 1 and (**b**) Test 2.

The completion rate graphs shown in Figure 7 further clarify these trends.

In Test 1 (Figure 7a), the Control group maintains a high completion rate throughout, while a significant drop in participation occurs in the Dyslexia without support group, particularly after Task 7, with only a small percentage completing the final tasks. The Dyslexia with support group exhibits a more gradual decline, with a noticeable drop starting at Task 9. In Test 2 (Figure 7b), the pattern is similar but with a steeper decline, particularly in the Dyslexia without support group, where task completion decreases more rapidly. Notably, in the Dyslexia without support group, no participant completed tasks beyond Task 8. This suggests that Test 2 was overall more challenging, leading to greater dropout rates, especially in participants without reading assistance. The lower completion rates in these groups align with the increased cognitive load observed in Test 2, as those who struggled the most may have disengaged earlier. The fact that the Control group had consistently higher completion rates, even in the later tasks, supports the idea that cognitive load was not a barrier for them, whereas, for the Dyslexia without support group, the increasing cognitive demands likely contributed to task abandonment.

### 5.2. Human–Robot Assistance Results

**Figure 8:**
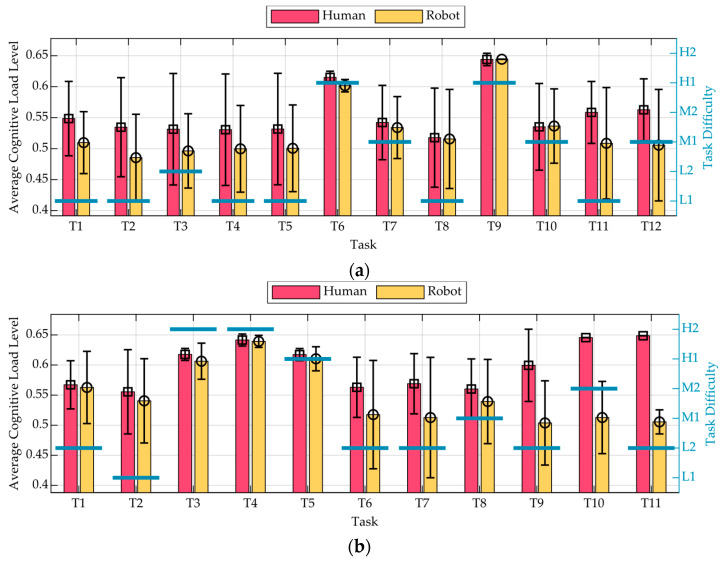
Average cognitive load levels per task and assistance conditions (Human, Robot) for the Reading output in (**a**) Test 1 and (**b**) Test 2. Vertical black error bars indicate the standard deviation. Task difficulty levels are shown as horizontal blue lines, labelled from L1 (Low 1) to H2 (High 2).

Building upon the prior analysis conducted across the three test scenarios, the CL data of the Reading fuzzy output were further examined by categorising participants based on the type of assistance received: Human Assistance and Robot Assistance. The CL signals were segmented into individual tasks: 12 for Test 1 and 11 for Test 2. For each task, the corresponding segment of the CL signal was extracted, and an average value was computed, resulting in a set of task-specific cognitive load values for each participant. Participants within the same assistance condition were then aggregated, and their task-specific average values were further averaged across individuals. The resulting charts, presented in Figure 8, illustrate how cognitive load varies across tasks depending on the type of assistance provided. Specifically, Figure 8a presents group-level means and standard deviations for each task in Test 1, corresponding to the Human Assistance and the Robot Assistance conditions. Similarly, Figure 8b reports results for Test 2. Vertical black error bars indicate the standard deviation, reflecting intra-group variability. Task difficulty was categorised into five levels, Low 1 (L1), Low 2 (L2), Medium 1 (M1), Medium 2 (M2), and High 1–2 (H1, H2), and is displayed using horizontal blue bars annotated with the corresponding labels.

Across both tests, participants supported by Human Assistance consistently exhibited higher CL levels compared to those assisted by a robot. In Test 1 (Figure 8a), this difference is particularly evident: the Human Assistance condition is associated with elevated CL levels across nearly all tasks, independent of task difficulty. Peaks in cognitive load are observed at Task 6 and Task 9, corresponding to the highest task difficulties; however, even during less demanding tasks, participants assisted by a human demonstrated higher cognitive load relative to those assisted by a robot. Standard deviation bars indicate greater variability in cognitive load within the Human Assistance condition across most tasks. This suggests that participants receiving human support exhibited more heterogeneous responses, possibly due to differing interpretations or interactions with the support provided. In contrast, Robot Assistance is associated with lower variability, reflecting a more consistent cognitive experience.

In Test 2 (Figure 8b), the trends of Human and Robot Assistance appear relatively aligned between tasks Task 1 and Task 5, both following the increase in task difficulty. However, from Task 6 onwards, a clear divergence emerges: Human Assistance CL levels progressively increase, while Robot Assistance CL levels remain comparatively stable. This indicates that, while both assistance modalities initially responded similarly to task complexity, human-assisted participants experienced a growing cognitive burden in the later stages of the test, whereas robot-assisted participants maintained lower and more consistent cognitive load levels. In Figure 8b, the standard deviation bars reveal that, unlike in Test 1, the Robot Assistance condition displays greater intra-group variability across several tasks. This suggests that, while robot-assisted participants maintained a lower average cognitive load, their individual responses were more dispersed. In contrast, the Human Assistance group exhibited more consistent levels of cognitive load, with smaller deviations from the mean values.

## 6. Conclusions

The present study proposed a methodology to assess cognitive load utilising physiological variables such as HR, ECG, HRV, EDA, and eye-related parameters, along with working environment and process-related variables. A fuzzy logic-based data analysis was adopted to estimate and detect variations in cognitive load across different assembly task scenarios. The results clearly indicated that participants with simulated dyslexia without support experienced higher cognitive load compared to scenarios in which assistance was provided, for higher task difficulty conditions. For relatively easy assembly tasks, the Control group showed unexpectedly higher cognitive load, possibly due to increased effort to maintain performance. Completion rates reflected these trends, with the Dyslexia without support group showing the steepest decline, particularly in more challenging scenarios. Additionally, participants assisted by a robot exhibited lower and more stable cognitive load compared to those supported by a human, suggesting that Robot Assistance may reduce cognitive demands across tasks.

Experimental test results emphasise the value of introducing personalised support within industrial settings to decrease cognitive stress and potentially improve the working efficiency of neurodiverse operators. Practical adaptations, such as assistive technologies including digital instructions and multimodal corrective guidelines, could meaningfully reduce mental workload, fostering a more inclusive and accessible environment.

Nevertheless, this study has certain limitations. The number of participants was defined in accordance with the constraints set by the ethical approval. Although the small sample size limits statistical generalisability and increases sensitivity to inter-individual differences in physiological signals, this study was designed as a methodological proof-of-concept. The main objective was to evaluate the feasibility of a multi-layer fuzzy logic framework for cognitive load estimation in inclusive manufacturing contexts. Furthermore, the use of a counterbalanced within-subject design helped mitigate order effects and improve the internal consistency of the results. Additionally, the precise replication of the experimental conditions in different industrial environments could pose challenges due to variability in equipment, sensor positioning, and individual physiological baselines. Furthermore, the use of neurotypical participants in a simulated dyslexia scenario reduces the ecological validity of the findings. The participation of neurodiverse individuals in experimental tests is, however, highly regulated, with ethical and institutional constraints making their direct involvement particularly challenging, especially in exploratory studies. State-of-the-art literature supports the use of simulated dyslexia conditions as a valid preliminary method to investigate cognitive load and evaluate support strategies under controlled settings, as detailed in Section 4.2. Additionally, the assembly task under investigation was purely manual; therefore, it was not possible to assess cognitive load related to the use of tools, which may play a significant role in real-world scenarios.

Future studies should involve larger, more diverse samples to enhance external validity and include participants with actual neurodivergent conditions to improve ecological validity. Exploring more complex, tool-based manufacturing tasks and longer durations would better reflect real industrial settings. Finally, the development of adaptive real-time monitoring systems could support the dynamic management of cognitive load, promoting more inclusive and responsive workplaces.

## Figures and Tables

**Table 1:** Experimental programme.

Participant	Pre-Test	Test 1	Test 2	Assistance
Participant 1	Baseline	Dyslexia with support	Control	Human
Participant 2	Baseline	Control	Dyslexia with support	Human
Participant 3	Baseline	Dyslexia without support	Control	Human
Participant 4	Baseline	Control	Dyslexia without support	Human
Participant 5	Baseline	Dyslexia with support	Dyslexia without support	Human
Participant 6	Baseline	Dyslexia without support	Dyslexia with support	Human
Participant 7	Baseline	Dyslexia with support	Dyslexia with support	Human
Participant 8	Baseline	Dyslexia without support	Dyslexia without support	Human
Participant 9	Baseline	Control	Control	Human
Participant 10	Baseline	Dyslexia with support	Control	Robot
Participant 11	Baseline	Control	Dyslexia with support	Robot
Participant 12	Baseline	Dyslexia without support	Control	Robot
Participant 13	Baseline	Control	Dyslexia without support	Robot
Participant 14	Baseline	Dyslexia with support	Dyslexia without support	Robot
Participant 15	Baseline	Dyslexia without support	Dyslexia with support	Robot
Participant 16	Baseline	Dyslexia with support	Dyslexia with support	Robot
Participant 17	Baseline	Dyslexia without support	Dyslexia without support	Robot
Participant 18	Baseline	Control	Control	Robot

**Table 2:** Task characteristics for Test 1, including the number of items to be assembled, the number of operations required, and task difficulty.

Task Number	Number of Items to Be Assembled	Number of Operations	Task Difficulty
Task 1	3	6	Low
Task 2	1	2	Low
Task 3	1	2	Low
Task 4	1	2	Low
Task 5	1	2	Low
Task 6	4	8	High
Task 7	3	5	Medium
Task 8	0	1	Low
Task 9	6	10	High
Task 10	0	2	Medium
Task 11	1	2	Low
Task 12	1	2	Medium

**Table 3:** Task characteristics for Test 2, including the number of items to be assembled, the number of operations required, and task difficulty.

Task Number	Number of Items to Be Assembled	Number of Operations	Task Difficulty
Task 1	4	8	Low
Task 2	2	4	Low
Task 3	4	8	High
Task 4	6	12	High
Task 5	4	8	High
Task 6	1	2	Low
Task 7	1	2	Low
Task 8	3	5	Medium
Task 9	0	1	Low
Task 10	2	2	Medium
Task 11	3	6	Low

## Data Availability

Data are available on request.
